# Inactivation of DNA–Dependent Protein Kinase Promotes Heat–Induced Apoptosis Independently of Heat–Shock Protein Induction in Human Cancer Cell Lines

**DOI:** 10.1371/journal.pone.0058325

**Published:** 2013-03-11

**Authors:** Seisuke Okazawa, Yukihiro Furusawa, Ayako Kariya, Mariame Ali Hassan, Mie Arai, Ryuji Hayashi, Yoshiaki Tabuchi, Takashi Kondo, Kazuyuki Tobe

**Affiliations:** 1 First Department of Internal Medicine, Graduate School of Medicine Pharmaceutical Sciences, University of Toyama, Toyama, Japan; 2 Department of Radiological Sciences, Graduate School of Medicine Pharmaceutical Sciences, University of Toyama, Toyama, Japan; 3 Department of Pharmaceutics and Industrial Pharmacy, Faculty of Pharmacy, Cairo University, Cairo, Egypt; 4 Division of Molecular Genetics Research, Life Science Research Center, Graduate School of Medicine Pharmaceutical Sciences, University of Toyama, Toyama, Japan; Boston University Medical School, United States of America

## Abstract

The inhibition of DNA damage response pathway seems to be an attractive strategy for cancer therapy. It was previously reported that in rodent cells exposed to heat stress, cell growth was promoted by the activity of DNA-dependent protein kinase (DNA-PK), an enzyme involved in DNA non-homologous end joining (NHEJ) required for double-strand break repair. The absence of a functioning DNA-PK was associated with down regulation of heat shock protein 70 (HSP70). The objective of this study is thus to investigate the role of DNA-PK inhibition in heat-induced apoptosis in human cell lines. The inhibitors of phosphorylation of the DNA-PK catalytic subunit (DNA-PKcs) at Ser2056, such as NU7026 and NU7441, were utilized. Furthermore, knock down of DNA-PKcs was carried out using small interfering RNA (siDNA-PKcs). For heat exposure, cells were placed in water bath at 44°C for 60 min. Apoptosis was evaluated after 24 h incubation flow cytometrically. Proteins were extracted after 24 h and analyzed for HSP70 and HSP40 expression by Western blotting. Total RNA was extracted 6 h after treatment and analyzed using a GeneChip® microarray system to identify and select the up-regulated genes (≥1.5 fold). The results showed an enhancement in heat-induced apoptosis in absence of functioning DNA-PKcs. Interestingly, the expression levels of HSP70 and HSP40 were elevated in the absence of DNA-PKcs under heat stress. The results of genetic network analysis showed that HSPs and JUN genes were up-regulated independently of DNA-PKcs in exposed parent and knock out cells. In the presence of functioning DNA-PKcs, there was an observed up-regulation of anti-apoptotic genes, such as NR1D1, whereas in the absence of DNA-PKcs the pro-apoptotic genes, such as EGR2, were preferentially up-regulated. From these findings, we concluded that in human cells, the inactivation of DNA-PKcs can promote heat-induced apoptosis independently of heat-shock proteins.

## Introduction

Malignant tumors are a major problem in the world that in the medical research field the developing of effective therapeutics has always been on the top of research interests for decades. This goal has persisted over years despite the numerous therapeutic tools available (such as surgery, chemotherapy, radiotherapy, hyperthermia [1], high intensity focused ultrasound (HIFU) [2], immunotherapy [3], etc.), and the variety of their strategic combinations [1], [4], [5]. This is because none of these tools has worked perfectly or safely in eradicating cancers. However, to set scientific grounds to the discoveries of novel approaches, a thorough understanding of cancer molecular biology becomes mandatory. Recently, the studies on DNA damage response (DDR) pathways rendered this area as promising in cancer treatment. The combination of DNA-damaging agents with molecular targeting drugs against the players involved in DNA repair pathways could result a synergistic effect in killing cancer cells [6]. The studies on DDR revealed a plethora of molecular targets such as the Ataxia telangiectasia mutated (ATM), Ataxia telangiectasia and Rad3-related (ATR), and DNA-dependent protein kinase (DNA-PK). These proteins are members of the phosphoinositol 3-kinase-like kinase (PIKK) family functioning as transducers in DDR to activate multiple proteins involved in cellular response to DNA damage [7]–[9].

It was reported that DNA-PK interacts with heat shock transcription factor (HSF1) [10]. In one study, the DNA-PKcs negative mouse cell line scva2 and a matched DNA-PKcs positive cell line sc(8)-10 derived from scva2 by the introduction of a DNA-PKcs structural gene, were exposed to heat treatment and the extent of heat-induced apoptosis was evaluated. The DNA-PKcs negative cells showed somewhat delayed HSP70 induction which reached a lower steady state level. This observation was justified by HSF1 and DNA-PKcs interaction [11]. However, as DDR activation by hyperthermia was not recognized at that time, the involvement of ATM activation, p53 phosphorylation [12], and the induction of γH2AX [13] by heat stress was not reported. Recently, the phosphorylation of p53 at Ser15 as well as the intracellular signaling of the non-homologous end joining (NHEJ) has been revealed. In addition, several other scenarios of DNA-PK involvement in heat-stress response, such as cell survival signal up-regulation by Akt phosphorylation through DNA-PK [14] and activation of NFκB p50 [15], have been also reported. Furthermore, our group has shown that the inhibition of DNA-PK by DNA-PK inhibitors and RNAi technique promoted ultrasound-induced cell death regardless of p53 phenotype [16].

In this study, we will similarly investigate the role of DNA-PK in heat-induced apoptosis in different human cancer cell lines. We hypothesize that the DNA-PKcs inhibition might reinforce hyperthermia-induced cell death. The details of the underlying mechanisms will be also studied.

## Results and Discussion

### Heat-induced Apoptosis was Enhanced by DNA-PK Inhibitors

**Figure 1:**
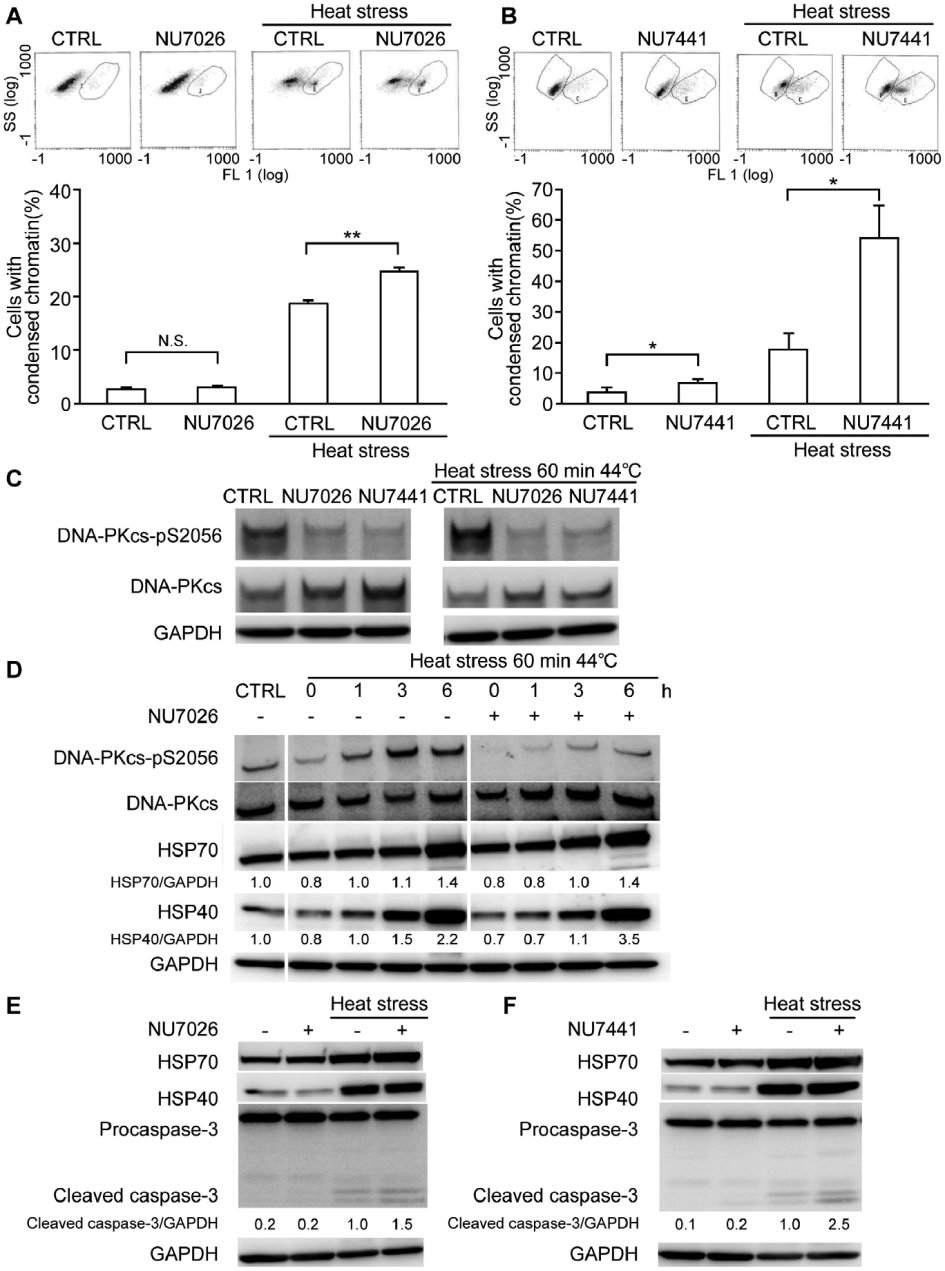
DNA-PK inhibitors increased heat-induced apoptosis independently of HSPs in HeLa cells. Chromatin condensation in cells in the presence of 10 µM of (a) NU7026, or (b) NU7441. (c) The inhibition of DNA-PKcs phosphorylation at Ser2056 by 10 µM of NU7026 or NU7441 6 h post exposure to heat stress. (d) Time-dependent Western blot analysis of HeLa cells (0–6 h) showing the changes in the phosphorylation of DNA-PKcs at Ser2056 (DNA-PKcs-pS2056) and HSPs; HSP70 and HSP40, by 10 µM of NU7026. Caspase-3 cleavage and HSP expression by Western blotting 24 h after treatment in the absence or presence of 10 µM of (e) NU7026 or (f) NU7441. NU7441 induced weak caspase-3 activation without heat exposure in augmentation with chromatin condensation data. Data are expressed as the means ± SD (*, P<0.05; **, P<0.01; N.S., non-significant).

Control and treated cells were analyzed for the extent of chromatin condensation as an indicator for apoptosis induction using a Nuclear-ID™ green chromatin condensation kit measured flow cytometrically. The analysis was performed 24 h after heat exposure in absence or presence of DNA-PK inhibitors. As shown in Figure 1A & B, both DNA-PK inhibitors NU7026 and NU7441 enhanced apoptosis significantly following heat stress. Interestingly, NU7026 had no effect on cells under normal conditions whereas NU7441 induced significant apoptosis in HeLa cells in absence of heat stress. This was probably due to the higher potency of the later against other members of the PIKK family proteins such as mammalian target of rapamycin (mTOR) and phosphatidylinositide-3-kinase (PI3K) [17]. It should be denoted here that DMSO was confirmed not to interfere with the results obtained (data not shown).

In Western blotting, Figure 1C shows that both inhibitors could successfully suppress the phosphorylation of DNA-PKcs at Ser2056 at 6 h post exposure to heat stress. Time-dependent analysis of cells showed that the phosphorylation of DNA-PKcs at Ser2056 increased gradually with time up to 6 h after heat treatment. In the same time course, there was a simultaneous increase in the expression of HSP70 and HSP40. Strikingly, upon DNA-PK inhibition, DNA-PKcs-pS2056 decreased while HSP70 and HSP40 expression retained its time-dependent increase (Figure 1D). The retention of HSPs increase pattern in absence of DNA-PK comes in contrast to the associated decrease reported in mouse cells and may thus reflect that the sensitization of human cells to apoptosis through DNA-PK inhibition might operate independently of HSPs. To prove this special difference, we confirmed that 10 µM of NU7441 treatment decreased the expression of HSP70 in Chinese hamster ovarian cells, CHO-K1 (Figure S1).

Cell death through apoptosis induction was further confirmed by caspase-3 cleavage. In Figure 1E & F, caspase-3 cleavage was detected 24 h post heating. Caspase-3 cleavage was significantly enhanced in presence of inhibitors especially NU7441. NU7441 treatment induced weak band in non-heated cells in augmentation with chromatin condensation data. Moreover, the data reveals that the increase in HSP70 and HSP40 was sustained up to 24 h post treatment with DNA-PK inhibitor.

### Heat-induced Apoptosis was Enhanced by DNA-PKcs Knockdown Using Small Interference RNA

**Figure 2:**
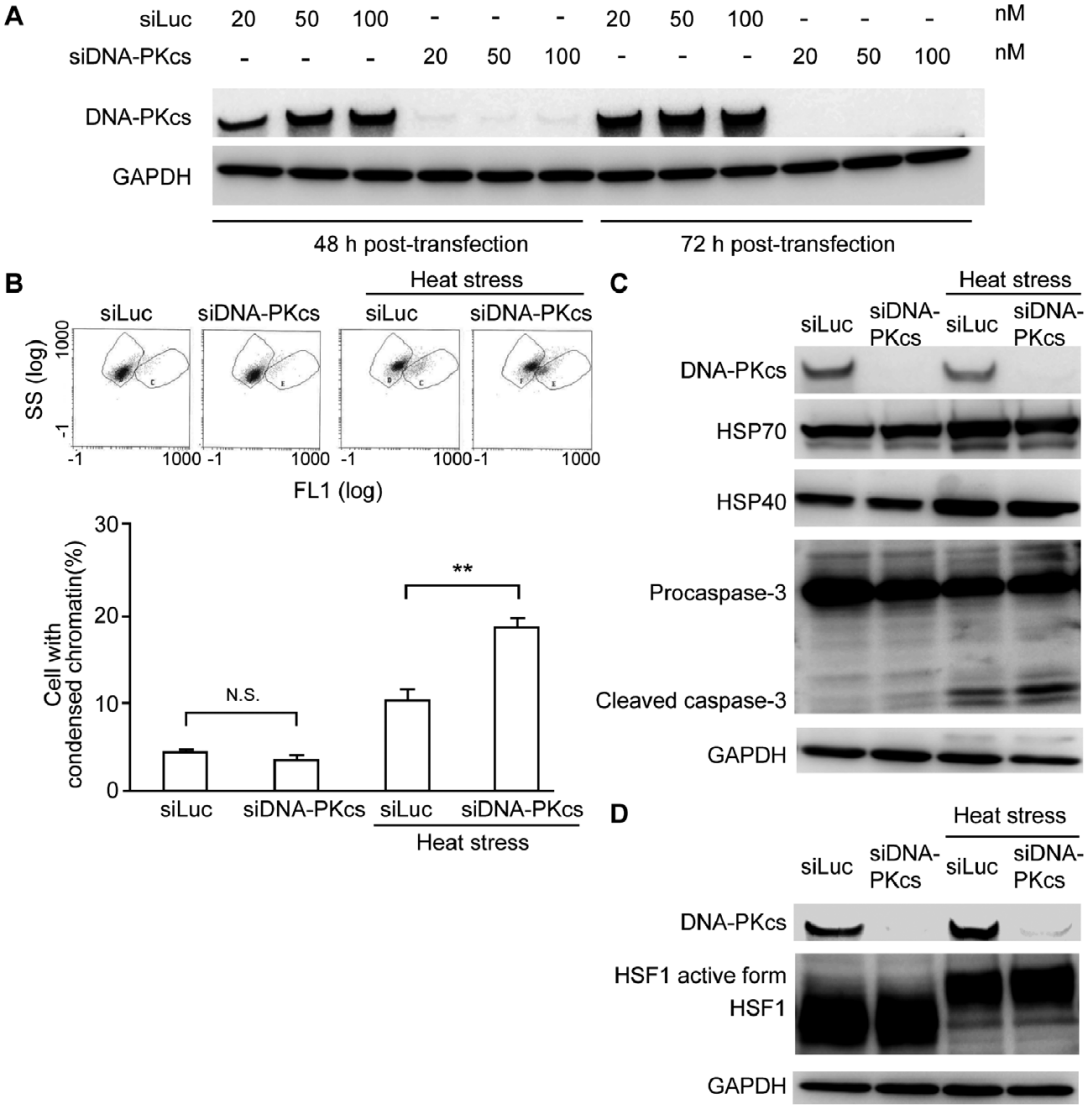
DNA-PKcs knockdown increased the heat-induced apoptosis independently of HSPs in HeLa cells. (a) HeLa cells transfected by different doses of small interference RNA against DNA-PKcs (siDNA-PK) and luciferase (siLuc); (b) DNA-PKcs- knockdown cells exposed to heat stress showed higher percentage of chromatin condensation; (c) The knockdown of DNA-PKcs enhanced caspase-3 activation following heat stress independently of HSP70 and HSP40 expression at 24 h post treatment; (d) HSF1 active form band was enhanced in absence of DNA-PKcs 1 h after exposure to heat stress. Data are expressed as the mean ± SD (**, P<0.01, N.S.; non-significant).

In order to confirm the role of DNA-PK in heat-induced apoptosis, we performed alternative experiments on cells lacking a functional DNA-PK through silencing the protein by a DNA-PKcs-targeted siRNA (siDNA-PKcs). The transfection procedures were first confirmed by Western blotting 48 and 72 h following transfection. Figure 2A shows the absence of endogenous DNA-PKcs bands at all concentrations of siDNA-PKcs. Further experiments were thus conducted using cells transfected at 20 nM. Cells transfected with luciferase siRNA (siLuc, 20 nM) under similar conditions were taken as negative controls. Upon exposure to heat, cells lacking functional DNA-PK displayed significant chromatin condensation compared to cells transfected with siLuc (Figure 2B). In support, the cleaved caspase-3 bands 24 h post treatment increased significantly in siDNA-PKcs-transfected cells. These results further support the role of DNA-PKcs in heat-induced apoptosis. Again, there was no obvious relation among HSPs and DNA-PK silencing after exposure to heat stress (Figure 2C). This finding comes in contrast to reports on rodent cells lacking functional DNA-PK showing that the expression level of HSP70 under heat stress was down-regulated [11] (Figure S2). To confirm the generalization to other human cells, we carried out similar experiments on two variants of malignant glioma cells, namely; M059K cells with functioning DNA-PK and the DNA-PKcs defective M059J variant. As shown in Figure S3, the expression of HSP70 and HSP40 was similar in both cells regardless of DNA-PK status. As such, it might be concluded that in human cells, the inhibition of DNA-PK enhances heat-induced apoptosis independently of HSPs.

The expression of HSF1 active form was analyzed in HeLa cells at 1 h post heat exposure (44°C for 60 min). HSF1 active form was up-regulated in absence of DNA-PKcs (Figure 2D). This finding reflects that HSF1 activation does not require DNA-PKcs.

### Gene Expression Analysis

**Figure 3:**
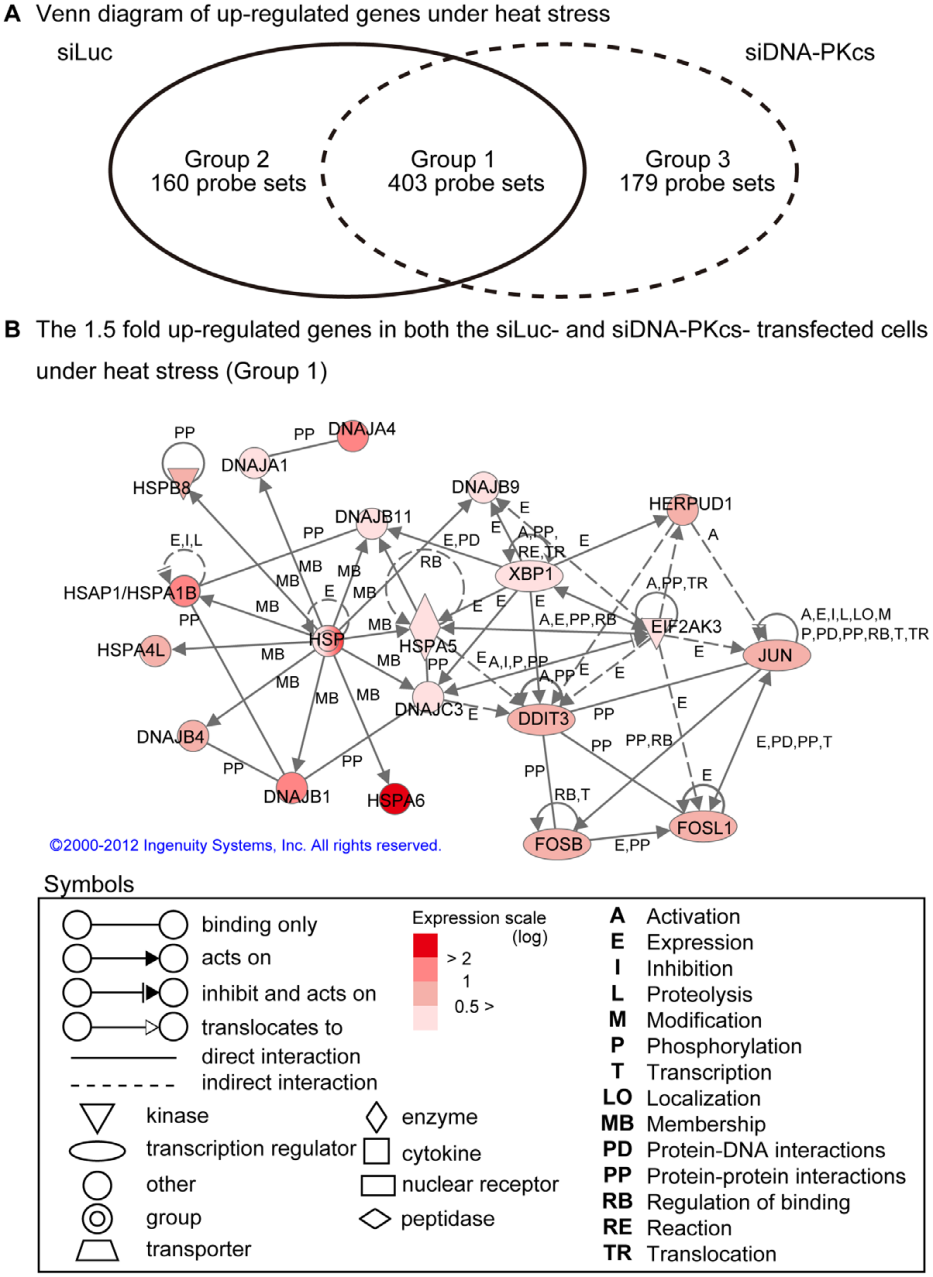
Genetic network of genes up-regulated independently of DNA-PK following heat stress. (a) Illustrative Venn diagram of up-regulated genes in siLuc- and siDNA-PKcs-transfected cells after exposure to heat stress; (b) Common up-regulated genes in both siLuc- and siDNA-PKcs-transfected cells exposed to heat stress (Group 1); the genetic network is displayed graphically as nodes (genes) and edges (the biological relationships between genes). The node color indicates the expression level of the respective gene. Nodes and edges are displayed in various shapes and labels that represent the functional classes of genes and the nature of the relationship among nodes, respectively. This network shows that HSP genes were not associated with DNA-PK status and that JUN-related genes were up-regulated in response to heat-induced cell damage.

Figure 3A shows that heat stress could up-regulate 403 probe sets by ≥1.5 fold in both the siLuc- and siDNA-PKcs-transfected cells (group 1). Another 160 probe sets were up-regulated under heat stress by ≥1.5 fold in siLuc-transfected cells only (group 2) whereas 179 probe sets were up-regulated by ≥1.5 fold in siDNA-PKcs-transfected cells (group 3). The genetic network that comprises group 1 genes showed that HSPs, such as HSP70 (HSPA6) and HSP40 (DNAJB1), were highly expressed (Figure 3B). Pro-apoptotic genes as jun proto-oncogene (JUN) and FBJ murine osteosarcoma viral oncogene homolog B (FOSB) were also identified as up-regulated genes. The up-regulation of group 1 genes in both cell variants suggests that these genes respond to heat stress independently of DNA-PK status, the thing that further supports the Western blot data. Similar to HeLa cells, our previous studies showed that the expression of the basic-region leucine zipper (bZIP) transcription factors JUN, ATF3 and FOS were also enhanced in human lymphoma U937 cells showing apoptosis after exposure to heat stress at 44°C for 15 min [18].

**Figure 4:**
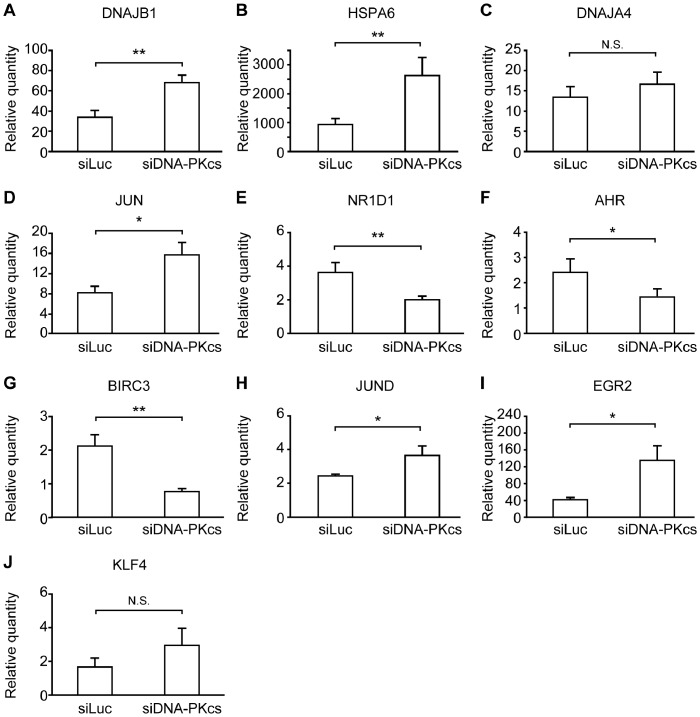
Verification of microarray results by real-time qPCR. HeLa cells were treated at 44°C for 60 min and then incubated at 37°C for 6 h. The mRNA expression levels of (a) DNAJB1 (HSP40), (b) HSPA6 (HSP70), (c) DNAJA4, (d) JUN, (e) NR1D1, (f) AHR, (g) BIRC3, (h) JUND, (i) EGR2 and (j) KLF4 normalized to GAPDH expression level. Data are expressed as the mean ± SD (*, P<0.05; **, P<0.01; N.S., non-significant).

The expression profile of some selected genes, namely; HSPA6, DNAJB1, DNAJA4 and JUN, was determined using real-time qPCR (Figure 4A–D). The results showed that the mRNA levels of these genes were significantly increased by heat stress.

**Figure 5:**
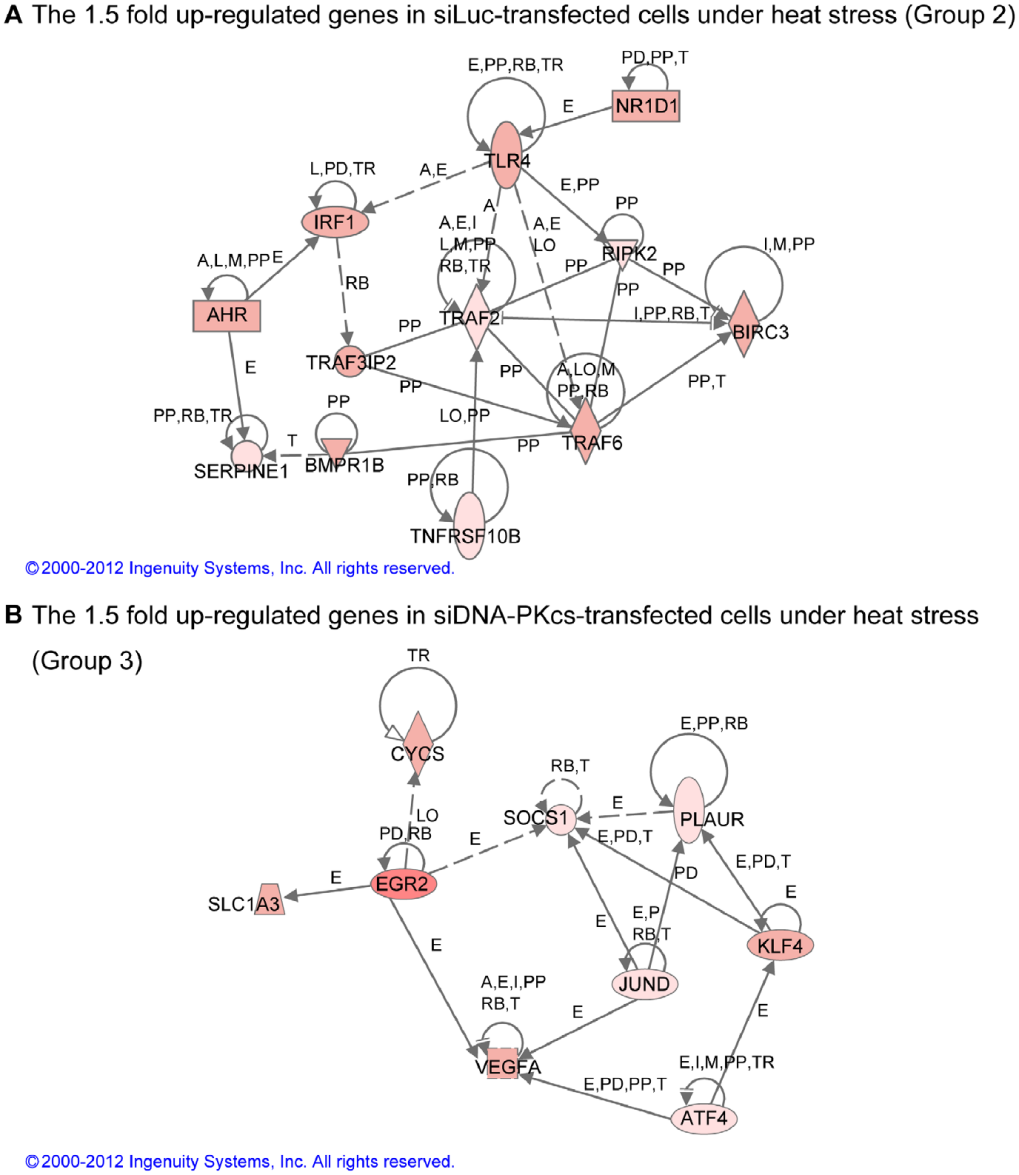
Genetic networks of genes up-regulated dependently on DNA-PK status following heat stress. (a) Up-regulated genes in heat-exposed siLuc-transfected cells (Group 2 in Fig. 3A). This group comprises anti-apoptotic genes such as NFκB pathway related genes, AHR and NR1D1; (b) Up-regulated genes in heat-exposed siDNA-PKcs-transfected cells (Group 3 in Fig. 3A). This group comprises pro-apoptotic genes that are normally inhibited by DNA-PK activity such as EGR2, KLF4 and ATF4.

Next, we depicted the possible genetic network which would comprise the up-regulated genes in siLuc-transfected cells (group 2) (Figure 5A). The genes identified included pro-apoptotic genes that work through TNFα-related pathways such as TLR4 [19], RIPK2 [20], and TNFRSF10B, also known as DR5, that can be activated by tumor necrosis factor-related apoptosis inducing ligand [21]. The tumor necrosis factor receptor-associated factors, such as TRAF2 and TRAF6, were also detected. TRAF2 mediate signaling from the TNF-R superfamily for the activation of JNK (c-Jun N-terminal Kinase) or NFκB [22]. TRAF6 also participates in the signaling pathway from the TLR superfamily and induces apoptosis through a caspase-dependent pathway and increases NFκB activation [23]. TRAF3IPs interacts with TRAF family proteins and either I-κB kinase or MAP kinase [24]. IRF1 gene induces cell death in the presence of INFγ and TNFα [25]. On the other hand, some cell survival genes were also identified. The up-regulation of the survival genes was again confirmed by qPCR assay. Figure 4E shows that the mRNA level of NR1D1 was up-regulated in siLuc-transfected cells while down-regulated in DNA-PKcs knockdown cells. NR1D1 is depicted in the network as the gene that might influence TLR4. NR1D1 was reported to be an LXR target gene in human macrophages and represses LXR-induced-TLR-4 expression [26]. Interestingly, NR1D1 is one of the intranuclear receptors and is considered to contribute to the circadian system, however, the biological mode of its action is unknown. A recent report suggests NR1D1 may act on fat metabolism enzyme activity in human epidermal growth factor receptor 2 (ERBB2)-positive breast cancer, and to function in cell survival [27], [28]. Thus, it could be that NR1D1 is induced under heat stress by DNA-PK to promote cell metabolism for survival. Similarly, Figure 4F & G show the differential expression of AHR and BIRC3 in both cell variants. AHR has many functions in ATM signaling, bone differentiation, Th17 lymphocyte differentiation, P450 expression, and the regulation of the NFκB pathway [29], [30]. In addition, AHR enhances the cytoprotection-related gene SERPINE1 [31] which was also identified as an up-regulated gene in our previous studies [32], [33]. BIRC3 (cellular inhibitor of apoptosis protein 2; cIAP2) is one of the inhibitors to apoptosis family proteins. BIRC3 is induced by TNFα via NFκB pathway, and may be induced by other pathways including PI3K [34]. At this point, it was important to confirm the role of these survival genes in rescuing cells after heat stress exposure. For this, we performed knockdown experiments against NR1D1 and BIRC3 in HeLa cells followed by heat treatment. The efficiency of knock down was verified by qPCR analysis 24 h following transfection (Figure S4). Transfected cells were exposed to heat stress 24 h after transfection. Western blot analysis showed that the cleaved caspase-3 levels increased slightly in siNR1D1- and siBIRC3-transfected cells 24 h post treatment (Figure S5). Cells lacking NR1D1 displayed significant increase in chromatin condensation compared to cells transfected with siLuc, whereas the knockdown of BIRC3 slightly, but not significantly, increased the percentage of cells with condensed chromatin following heat treatment (Figure S6).

Figure 5B illustrates the possible genetic network which would comprise the up-regulated genes in siDNA-PKcs-transfected cells (group 3). Figure 4H–J provide the qPCR results for some selected genes, namely, JUND, ERG2 and KLF4. Among the up-regulated genes is JUND which behaves similarly to JUN proteins to transactivate an AP-1-responsive promoter in conjunction (Figure 4H). JUND exhibits a cell-dependent role in preventing cell death in UV-stressed mouse embryonic fibroblasts [35], while enhancing UV-induced caspase-3 activity in human myeloblastic leukemia [36]. SOCS1 expression is induced by TLR stimulation and inhibits JAK/STAT signaling [37] as negative feedback model [38]. SOCS1, which is regulated by JUND in T-cells [39], regulates the duration of NFκB signaling by decreasing the expression of NFκB-dependent genes [40]. PLAUR is a urokinase-type plasminogen activator receptor gene. PLAUR regulates endothelial cell survival and is important for VEGF-induced anti-apoptosis [41]. The over expression of JUND leads to the down-regulation of VEGFA mRNA [42].

The genetic network also confirms the up-regulation of several pro-apoptotic genes which goes in coordination with the observed enhancement in apoptosis in absence of DNA-PK. EGR2 plays a key role in the PTEN-induced apoptotic pathway. ERG2 was differentially increased in siDNA-PK knock down cells (Figure 4I). The over expression of EGR2 induces apoptosis in various tumor cell lines via BNIP3L and BAK [43]. CYCS (cytochrome c gene which induces apoptosis through bcl-2 pathway upon its release from mitochondria to cytoplasm), activating transcription factor 4 (ATF4) (aids in the up-regulation of the pro-apoptotic protein C/EBP-homologous protein expression [44]), and the zinc-finger transcription factor KLF4 (reported to be involved in apoptosis induction in T-cell leukemia [45]) were all identified as up-regulated genes. The increase in mRNA level of KLF4 was below the significance level (p = 0.078), however, the tendency was discernible in all replicates (Figure 4J). These pro-apoptotic genes might be inhibited by DNA-PK activity and thus, in absence of a functional DNA-PK, these genes may lead to an enhancement of heat-induced apoptosis.

## Materials and Methods

### Chemicals

The selective DNA-PK inhibitor NU7026 (2-(morpholin-4-yl)-benzo[h]chomen-4-one) was purchased from Sigma-Aldrich (St Louis, MO) and NU7441 (8-(4-Dibenzothienyl)-2-(4-morpholinyl)-4H-1-benzopyran-4-one) was purchased from Axon Medchem (Groningen, The Netherlands). They were dissolved in dimethyl sulfoxide (DMSO) at a final concentration of 10 mM. Stock solutions were stored at -20°C until use.

### Cell Lines and Treatment

Human cervical carcinoma HeLa S3 cells (Riken, Tsukuba, Japan), human malignant glioma M059K and its DNA-PKcs defective variant M059J (provided by A. Takahashi, Gunma University, Japan), were all grown in DMEM supplemented with 10% fetal bovine serum (FBS) and 1% penicillin/streptomycin antibiotic mixture. And Chinese hamster ovary cell CHO-K1 and its DNA-PKcs defective variant V3 cells [46] (Riken) were all grown in MEMα supplemented with 10% FBS and 1% penicillin/streptomycin antibiotic mixture. Heating was performed by immersing a parafilm-sealed cell culture plate in a water bath at 44°C for 60 min. The temperature was monitored with a digital thermometer (# 7563, Yokogawa, Tokyo, Japan). In inhibition experiments, cells were pre-incubated with 10 µM of NU7026 or NU7441 for 30–60 min, followed by heat exposure. Treated cells were then incubated at 37°C until analysis without medium exchange.

### Chromatin Condensation Analysis

Control and treated cells were collected 24 hr after treatment and stained using a Nuclear-ID™ green chromatin condensation detection kit (Enzo Life Sciences, Farmingdale, NY) according to the manufacturer’s protocol. The extent of chromatin condensation was measured by calculating the fluorescence intensity of the Nuclear-ID™ green relative to control with flow cytometry (Epics XL, Beckman Coulter, Miami, FL).

### Western Blotting Analysis

Cells were dissolved in lysis buffer (50 mM NaCl, 1% Nonidet P-40 and 50 mM Tris-HCl, pH 8.0) containing sodium orthovanadate and proteases inhibitor cocktail (NACALAI TESQUE, Kyoto, Japan). SDS-polyaclylamidegel electrophoresis and Western blotting were carried out as described elsewhere [47]. The primary antibodies used were as follows: a mouse monoclonal anti-HSP70 antibody (MBL, Nagoya, Japan); a mouse monoclonal anti-HSP40 antibody (MBL); a rabbit polyclonal anti-caspase 3 antibody which recognizes the full length caspase-3 (35-kDa) as well as the fragments (19- and 17-kDa) of the activated enzyme (Cell Signaling Technology, Danvers, MA); a rabbit polyclonal anti-phospho DNA-PKcs at Ser2056 antibody (Abcam, Cambridge, UK); a rabbit monoclonal anti-DNA-PKcs antibody (Epitomics, Burlingame, CA); a rabbit polyclonal anti-HSF1 antibody (Stressgen Bioreagents, Victoria, Canada); and a mouse monoclonal anti-glyceraldehyde 3-phosphate dehydrogenase (GAPDH) antibody (Organon Teknika, Durham, NC). Immunoreactive proteins were visualized using enhanced chemiluminescence (ECL) detection system on a luminescent image analyzer (LAS-4000, Fujifilm, Tokyo, Japan).

Band densities were quantified by the Image J (Rasband, W.S., ImageJ, U. S. National Institutes of Health, Bethesda, Maryland, USA, http://imagej.nih.gov/ij/, 1997–2012.) and the relative amounts of proteins associated with each specific antibody were normalized to GADPH bands.

### Transfection with Small Interfering RNA (siRNA)

Negative control luciferase siRNA and DNA-PKcs-targeted siRNA were obtained from Nippon Gene Material (Toyama, Japan), and Thermo Fisher Scientific, Dharmacon Products (Lafayette, CO), respectively. The siRNAs targeting NR1D1 (Rev-erbα) and BIRC3 (c-IAP2) were purchased from Santa Cruz Biotechnolgy, Santa Cruz, CA. Transfection was carried out in HeLa cells using RNAi Max (Invitrogen, Carlsbad, CA) according to the manufacture’s protocol. In brief, HeLa cells were collected, washed and suspended in complete growth medium without antibiotics. The cells were seeded at a density of 2.5×10^4^ cells/well in 12-well plates and left to attach for 24 h. For transfection, 800 µl of Opti-MEM (Invitrogen) were added to cells followed by 200 µl of Opti-MEM containing the RNAi Max/siRNA complexes prepared 20 min before addition (Reverse transfection protocol). Six hours later, the transfection medium was replaced with fresh complete medium without antibiotics. Then the cells were incubated further 42 h. Thirty minutes before heat treatment, the medium was exchanged with complete medium with antibiotics.

### RNA Isolation

Total RNA was extracted from cells using RNeasy mini kit (Qiagen, Valencia, CA) and treated with DNase I (RNase-Free DNase kit; Qiagen) for 15 min at room temperature to remove residual genomic DNA. RNA quality was analyzed using a Bioanalyzer 2100 (Agilent Technologies, Inc., Santa Clara, CA). RNA samples that had RNA integrity number (RIN) values above 9.5 were considered acceptable.

### Microarray and Computational Gene Expression Analyses

Gene expression was analyzed using a GeneChip® Human Gene 1.0 ST Array spotted with approximately 28,869 probe sets (Affymetrix, Santa Clara, CA). Samples for array hybridization were prepared as described in the Affymetrix GeneChip® Expression Technical Manual. The scanned arrays were analyzed using the GeneChip® Analysis Suite Software (Affymetrix). The obtained hybridization intensity data were analyzed using the GeneSpring® analysis software ver 11.5 (Silicon Genetics, Redwood City, CA) to extract significant genes. To examine gene ontology, including the biological processes, cellular components, molecular functions, and genetic networks, the obtained data were analyzed using the Ingenuity Pathways Analysis tools (Ingenuity® Systems, Mountain View, CA, USA), a web-delivered application that enables the identification, visualization, and exploration of molecular interaction networks in gene expression data [48]. Complete lists of probe sets from all samples are available on the Gene Expression Omnibus (http://www.ncbi.nlm.nih.gov/geo/query/acc.cgi?acc=GSE39063).

### Real-time Quantitative Polymerase Chain Reaction (qPCR)

Real-time qPCR assay was performed on the Mx3000P® real-time PCR system (Stratagene Japan, Tokyo, Japan) using SYBR® Premix Ex Taq (Takara Bio, Shiga, Japan) according to the manufacturer’s protocol. Reverse transcriptase reaction (PrimeScript® reagent Kit, Takara Bio) was performed with DNase-treated total RNA. The primers were designed based on the following database sequences: NM 006145, DnaJ (HSP40) homolog, subfamily B, member 1 (DNAJB1); NM 002155, heat shock 70 kDa protein 6 (HSP70B’) (HSPA6); NM 018602, DnaJ (HSP40) homolog, subfamily A, member 4 (DNAJA4); NM 002228, jun proto-oncogene (JUN); NM 021724, nuclear receptor subfamily 1, group D, member 1 (NR1D1); NM 001621, aryl hydrocarbon receptor (AHR); NM 001165, baculoviral IAP repeat containing 3 (BIRC3); NM 005354, jun D proto-oncogene (JUND); NM 000399, early growth response 2 (EGR2); NM 004235, Kruppel-like factor 4 (KLF4); NM 002046, glyceraldehyde-3-phosphate dehydrogenase (GAPDH). GAPDH was used as control for normalization [49].

### Statistical Analysis

Data are presented as means ± SD. Statistical significance between any two data sets was determined using Welch’s t-test using R software (The R Foundation for Statistical Computing, version 2.15.1, free software available at http://www.R-project.org) with p values <0.05 regarded as significant.

## Supporting Information

Figure S1 **Western blot analysis of HSP70 expression in Chinese hamster ovarian (CHO-K1) cells.** The DNA-PK inhibitor, 10 µM of NU7441 decreased the expression of HSP70 in CHO-K1 cells 6 h after exposure to heat stress at 44°C for 60 min.
(TIF)

Figure S2 **Western blot analysis of HSP70 expression in rodent cells.** Two variants of Chinese hamster ovary cells, CHO-K1 cells with functional DNA-PK and V3 cells with defective DNA-PKcs were exposed to heat stress at 44°C for 60 min and then the extent of HSP70 expression was assayed 6 h later. HSP70 was down-regulated in absence of functional DNA-PK in V3 cells.
(TIF)

Figure S3 **Western blot analysis of HSP70 and HSP40 expression in human malignant glioma cell lines.** In two variants of malignant glioma cells, M059K cells with functioning DNA-PK and the DNA-PKcs defective M059J variant, the expression of HSP70 and HSP40 was similar in both cells regardless of DNA-PK status.
(TIF)

Figure S4 **Verification of knockdown efficacy of NR1D1 and BIRC3 by real-time qPCR.** HeLa cells were transfected by 50 nM of either siNR1D1 or siBIRC3. Transfected cells were incubated at 37°C for 24 h. The expression levels of mRNA of (a) NR1D1 and (b) BIRC3 normalized to GAPDH expression level. Data are expressed as the mean ± SD (**, P<0.01; N.S., non-significant).
(EPS)

Figure S5 **Western blot analysis of caspase-3 in siNR1D1 and siBIRC3 transfected cells following heat stress.** Western blot analysis showed that the cleaved caspase-3 bands 24 h post treatment increased in siNR1D1- and siBIRC3-transfected cells.
(TIF)

Figure S6 **Chromatin condensation analysis in siNR1D1 and siBIRC3 transfected cells following heat stress.** Upon exposure to heat stress, cells with abrogated NR1D1 displayed significant increase in chromatin condensation compared to cells transfected with siLuc, whereas abrogation of BIRC3 tended to increase the extent of chromatin condensation without displaying statistical significance. Data are expressed as the mean ± SD (*, P<0.05; N.S., non-significant).
(EPS)
