# Supplementary figures and images for: Inactivation of DNA–Dependent Protein Kinase Promotes Heat–Induced Apoptosis Independently of Heat–Shock Protein Induction in Human Cancer Cell Lines
Source: PLoS One. 2013 Mar 11;8(3):e58325. doi: 10.1371/journal.pone.0058325 (PMC3594312; doi:10.1371/journal.pone.0058325)

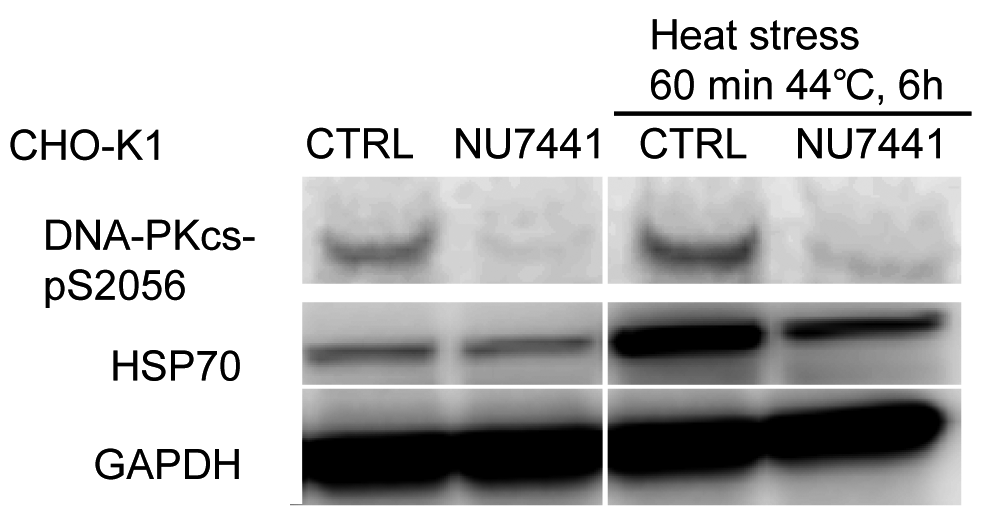

Supplement: Figure S1 — Western blot analysis of HSP70 expression in Chinese hamster ovarian (CHO-K1) cells. The DNA-PK inhibitor, 10 µM of NU7441 decreased the expression of HSP70 in CHO-K1 cells 6 h after exposure to heat stress at 44°C for 60 min. (TIF) [file pone.0058325.s001.tif]

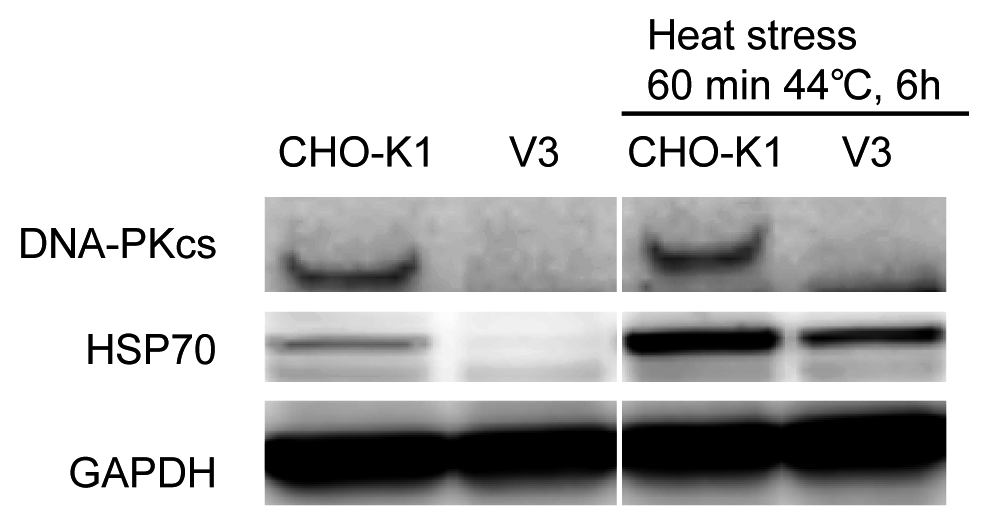

Supplement: Figure S2 — Western blot analysis of HSP70 expression in rodent cells. Two variants of Chinese hamster ovary cells, CHO-K1 cells with functional DNA-PK and V3 cells with defective DNA-PKcs were exposed to heat stress at 44°C for 60 min and then the extent of HSP70 expression was assayed 6 h later. HSP70 was down-regulated in absence of functional DNA-PK in V3 cells. (TIF) [file pone.0058325.s002.tif]

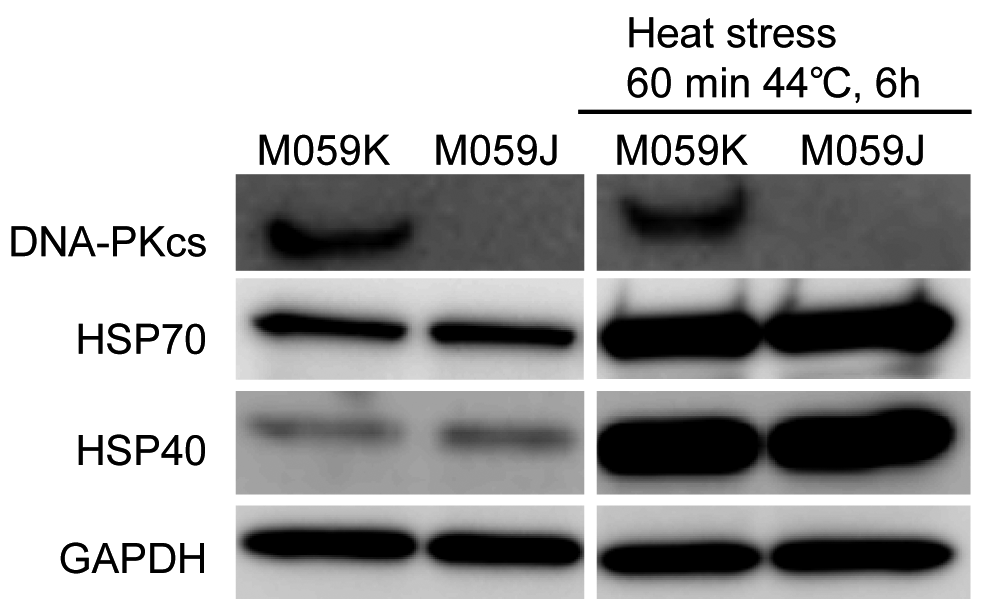

Supplement: Figure S3 — Western blot analysis of HSP70 and HSP40 expression in human malignant glioma cell lines. In two variants of malignant glioma cells, M059K cells with functioning DNA-PK and the DNA-PKcs defective M059J variant, the expression of HSP70 and HSP40 was similar in both cells regardless of DNA-PK status. (TIF) [file pone.0058325.s003.tif]

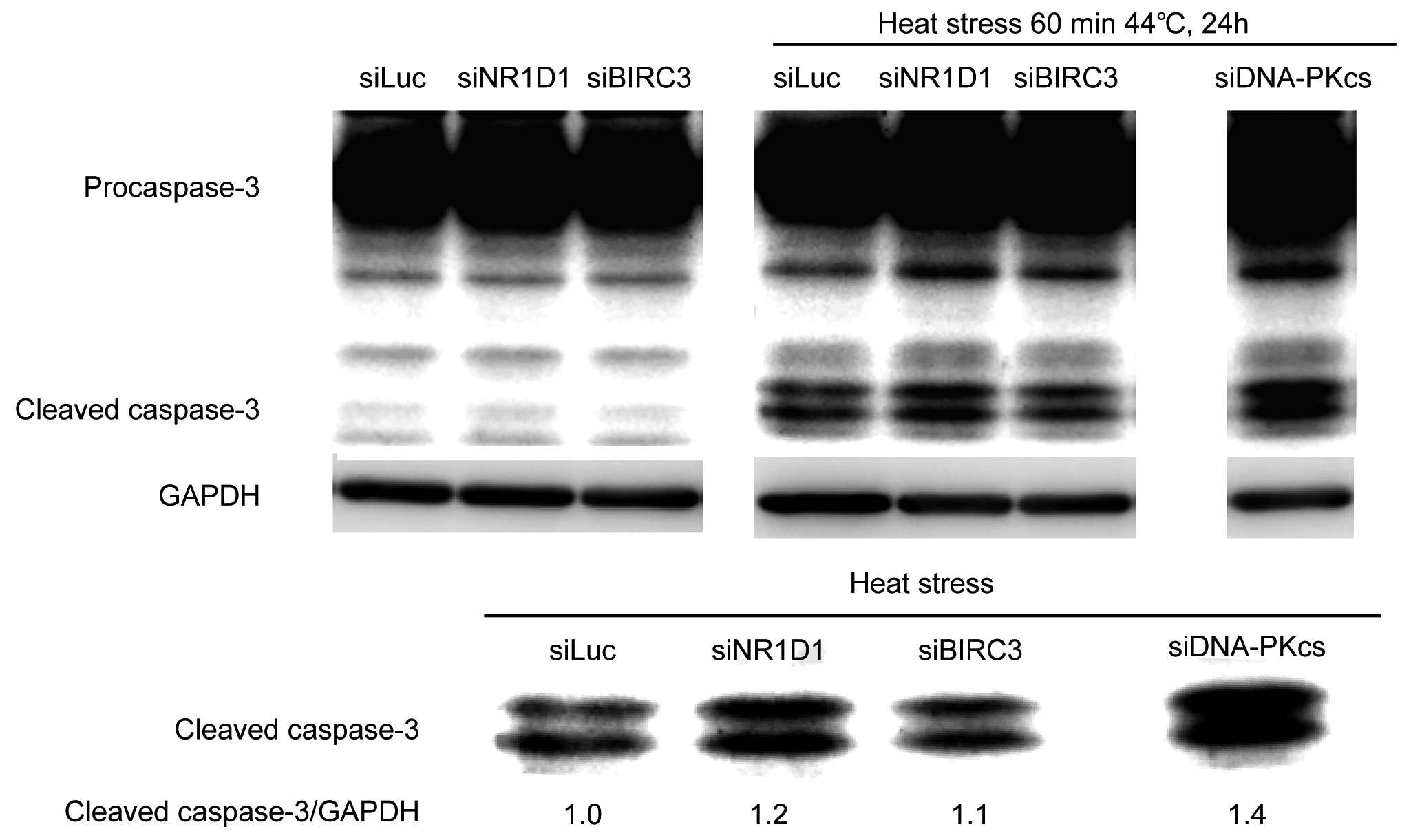

Supplement: Figure S5 — Western blot analysis of caspase-3 in siNR1D1 and siBIRC3 transfected cells following heat stress. Western blot analysis showed that the cleaved caspase-3 bands 24 h post treatment increased in siNR1D1- and siBIRC3-transfected cells. (TIF) [file pone.0058325.s005.tif]
